# Identification of NETs-related biomarkers and molecular clusters in systemic lupus erythematosus

**DOI:** 10.3389/fimmu.2023.1150828

**Published:** 2023-04-18

**Authors:** Haoguang Li, Xiuling Zhang, Jingjing Shang, Xueqin Feng, Le Yu, Jie Fan, Jie Ren, Rongwei Zhang, Xinwang Duan

**Affiliations:** Department of Rheumatology and Immunology, The Second Affiliated Hospital of Nanchang University, Nanchang, Jiangxi, China

**Keywords:** systemic lupus erythematosus (SLE), neutrophil extracellular traps (NETs), biomarker, bioinformatics, machine learning, clusters

## Abstract

Neutrophil extracellular traps (NETs) is an important process involved in the pathogenesis of systemic lupus erythematosus (SLE), but the potential mechanisms of NETs contributing to SLE at the genetic level have not been clearly investigated. This investigation aimed to explore the molecular characteristics of NETs-related genes (NRGs) in SLE based on bioinformatics analysis, and identify associated reliable biomarkers and molecular clusters. Dataset GSE45291 was acquired from the Gene Expression Omnibus repository and used as a training set for subsequent analysis. A total of 1006 differentially expressed genes (DEGs) were obtained, most of which were associated with multiple viral infections. The interaction of DEGs with NRGs revealed 8 differentially expressed NRGs (DE-NRGs). The correlation and protein-protein interaction analyses of these DE-NRGs were performed. Among them, HMGB1, ITGB2, and CREB5 were selected as hub genes by random forest, support vector machine, and least absolute shrinkage and selection operator algorithms. The significant diagnostic value for SLE was confirmed in the training set and three validation sets (GSE81622, GSE61635, and GSE122459). Additionally, three NETs-related sub-clusters were identified based on the hub genes’ expression profiles analyzed by unsupervised consensus cluster assessment. Functional enrichment was performed among the three NETs subgroups, and the data revealed that cluster 1 highly expressed DEGs were prevalent in innate immune response pathways while that of cluster 3 were enriched in adaptive immune response pathways. Moreover, immune infiltration analysis also revealed that innate immune cells were markedly infiltrated in cluster 1 while the adaptive immune cells were upregulated in cluster 3. As per our knowledge, this investigation is the first to explore the molecular characteristics of NRGs in SLE, identify three potential biomarkers (HMGB1, ITGB2, and CREB5), and three distinct clusters based on these hub biomarkers.

## Introduction

Systemic lupus erythematosus (SLE) is a multisystem autoimmune disease characterized by the appearance of a variety of autoantibodies, resulting in a wide range of tissue and organs damaged (1). It has a higher frequency in females than males, with a prevalence of 9: 1 (2). Global SLE prevalence in adults is estimated to range from 30-150/100,000 and an incidence of about 2.2-23.1/100,000 annually, that is more than five million people suffering this disease worldwide (3). Despite the increase in the SLE survival rate over the last several decades (4, 5), it is still linked with early mortality (6), indicating substantial medical challenges especially linked with diagnosis and treatment. The clinical presentation of SLE is highly heterogeneous and there is an increasing number of atypical cases, which poses challenges in diagnosing the disease. Moreover, SLE treatment usually involves broad-spectrum immunosuppressive therapy, the efficacy of which can vary considerably between individuals and may have significant adverse effects, particularly when taken for prolonged periods. Different etiology has resulted in distinct clinical manifestations and cellular and molecular bases for SLE. Therefore, it is of immense clinical importance to uncover the underlying pathogenesis of SLE for its diagnosis and management.

The pathogenesis of SLE is complex and associated with enhanced dysregulation of innate and adaptive immunity (7). Neutrophils, the most abundant innate immune cells in humans, have long been considered as non-specific at the front line of defense against infections, however, the elucidation of neutrophil extracellular traps (NETs) has drastically revolutionized our current knowledge about neutrophil’s activity and their significance during the immune response. NETs are extracellular networks composed of chromatin associated with cytosolic and granular proteins that are released by neutrophils in response to various stimuli, a process called NETosis (8–10). Recent research has highlighted that NETs are crucial for the stimulation and advancement of systemic autoimmune diseases and perform complex inflammatory responses that cause organ injury (11–14). For example, aberrant NETs production and/or its decreased clearance, as well as NETosis-associated molecule alterations, in both animals and humans suffering from lupus. The NETs secrete extruded nuclear antigens, these are the source of autoantigens, which also participate in the breakdown of self-tolerance during lupus. Increased NETs can also stimulate the release of pro-inflammatory cytokine interferon-a, produce a direct cytotoxic effect on different renal cells, and induce capillary necrosis and podocyte loss. Additionally, NETs can induce endothelial-mesenchymal trans-differentiation (EndMT), thereby, stimulating activated myofibroblasts and causing the production of extracellular matrix. Thus, NETs have also been suggested as a critical contributor to the development of SLE, and targeting NETs has also been shown to have therapeutic effects. However, a detailed and integrated investigation of NETs-associated genes in SLE remains to be conducted.

Because of significant advancements in gene microarray technology, researchers can now rapidly assess the expression levels of thousands of genes, which has contributed to increased genetic knowledge of the disease etiology. Therefore, this investigation employs bioinformatics tools to explore the molecular characteristics of NETs-related genes (NRGs) in SLE and identify reliable biomarkers and molecular clusters for better diagnosis and effective management.

## Materials and methods

### Data source

The GEOquery R package (15) was used to download microarray datasets relevant to SLE from the Gene Expression Omnibus (GEO) online database (16). The following filtering criteria were used: (1) they were all from SLE patients and healthy controls; (2) the sample size was relatively large, with more than 20 sample; (3) the test specimens were from humans; and (4) the tissues used for sequencing were whole blood (WB) or peripheral blood mononuclear cell (PBMC). Additionally, the data were available for free download from the GEO database. Based on the above criteria, four datasets (GSE45291, GSE81622, GSE61635, and GSE122459) relevant to SLE were finally included in this study. There were 312 samples in the GSE45291 dataset (GPL13158 platform), of which 292 were of SLE and 20 were of normal WB. This dataset was utilized as a training set for analysis. The GSE81622 dataset (GPL10558 platform) included 30 SLE and 25 normal PBMC samples, the GSE61635 dataset (GPL570 platform) had 99 SLE and 30 normal WB samples, and the GSE122459 dataset (GPL16791 and GPL18573 platforms) comprised 20 SLE and 6 normal PBMC samples. These three datasets were selected for validation analysis. Additionally, a total of 69 NRGs were obtained from a previous study (17) (**Supplementary Table S1**).

All raw data in our investigation was subjected to normalization and adjustment for background, and we also cross-referenced all probe names with their respective gene symbols. Additionally, we also used the “ComBat” algorithm (18) to address the batch effects to improve the efficacy of the subsequent analysis.

### Identification of differentially expressed genes

The adjust p-value < 0.05 and fold changes (FC) > 1.5 were used set as the cut-off criteria to evaluate DEGs between SLE and healthy controls *via* the R-program limma package (19), The data acquired was presented in volcano plot and heatmap with the help of the R-packages ggplot2 (20) and pheatmap (21), respectively.

### Functional annotation and pathway enrichment of DEGs

To determine DEGs’ role in SLE, clusterProfiler of the R-package (22) was used for Gene Ontology (GO) and Kyoto Encyclopedia of Genes and Genomes (KEGG) pathway analyses. The Benjamini-Hochberg multiple correction approach was used to compensate for FDR-adjusted (P < 0.05) data. The three categories; cellular component (CC), molecular function (MF), and biological process (BP) of the GO analysis were crucial for examining physiological functions (23). KEGG analysis was performed to investigate potential routes (24). The ggplot (20) and GOplot (25) R programs were used to plot the findings of GO and KEGG, respectively.

### Gene expression patterns and protein-protein interaction analyses of differentially expressed NRGs

Next, the expression patterns of the DE-NRGs were analyzed. First, the DE-NRGs were obtained through the interaction of DEGs with NRGs, and their chromosomal locations were revealed and visualized using Package RCircos (26). Thereafter, using the corrplot function (R package corrplot) (27) the correlations among the expression levels of DE-NRGs were confirmed. Furthermore, a PPI enrichment analysis was performed using the online software GeneMANIA (https://genemania.org/) (28), and any combined score > 0.4 interaction was deemed statistically significant. Gene interactions are represented as a network, where nodes stand in for individual genes and linkages for whole networks. To further elucidate the biological features and mechanism of interacting proteins of the DE-NRGs, functional annotation analysis of inter was performed using another web tool, Metascape (http://metascape.org/) (29).

### Identification of NETs-related hub gene based on machine learning algorithm

Following three machine learning algorithms were adopted, support vector machine (SVM), least absolute shrinkage and selection operator (LASSO), and random forest (RF) to screen hub genes from DE-NRGs, using R packages of glmnet (30), e1071 (31) and caret (32), and RF (33), respectively. LASSO logistic regression identified variables by looking for those with the lowest likelihood of classification mistakes (34). The SVM technique creates a hyperplane in the characteristic space with the maximum margin for isolating negative from positive instances (35). RF is a group of independent decision trees used in an ensemble machine-learning technique to predict regression or clustering (36). The optimal lambda for LASSO regression was selected based on 10 resampling iterations of 10-fold cross-validation. Furthermore, the performances of SVM and RF were also evaluated based on 10-fold cross-validations. The selection of hub SLE genes was based on the overlapping genes produced from the three algorithms.

### Construction and validation of a diagnostic model for SLE

With the R package of rms, multivariate logistic regression analysis was performed to establish a nomogram model based on the NETs-related hub genes (37) for the diagnosis of SLE. The “total points” reflect the total of the points assigned to the aforementioned predictors, whereas each predictor has a corresponding point. The receiver operating characteristic (ROC) curves and area under the curve (AUC) were obtained from both training and validation sets to confirm nomograms’ diagnostic value. ROC curves were assessed *via* the ROC R package (38). Moreover, the decision curve analysis (DCA) and calibration curve were applied to evaluate the accuracy and practical applicability of the diagnostic model with the rms R package (37).

### Consensus cluster analysis

With the help of the ConsensusClusterPlus R package (39), an unsupervised hierarchical clustering (50 iterations, resample rate of 80%) was performed on the 292 SLE samples from the training set based on the expression of the NETs-related hub genes. This clustering was based on: 1) the gradual and smooth increase of the cumulative distribution function (CDF) curve. 2) no small sample size in any group. 3) delta area should have the largest decrease. 4) after clustering, the intra-group correlation should increase, while the inter-group correlation should decrease.

### Functional distinctions among the sub-clusters

Afterward, the NETs-related sub-clusters were compared pairwise, and the highly expressed DEGs were determined according to statistical criteria (FC > 1.5 and adj.p < 0.05) in each cluster. Followed by that GO and KEGG enrichment analyses were carried out to depict their physiological activities and visualized with the circle (40) and GOplot (25) R packages, respectively.

### Evaluating the immune infiltration of the sub-clusters

To evaluate sub-clusters’ immune infiltration, the R packages of GSVA (41) and GSEAbase (42) were utilized to quantify the relative abundance of immune cells and immune-related pathways and then compared enrichment scores across the sub-clusters, the results of which were visualized using the ggpubr R package (43).

### Statistical analysis

Unless otherwise stated, all investigations and visualization were carried out *via* R software (version.4.2.1). *P* < 0.05 and FC > 1.5 were set as the criteria for statistical importance.

## Results

### Identification of DEGs

**Figure 1:**
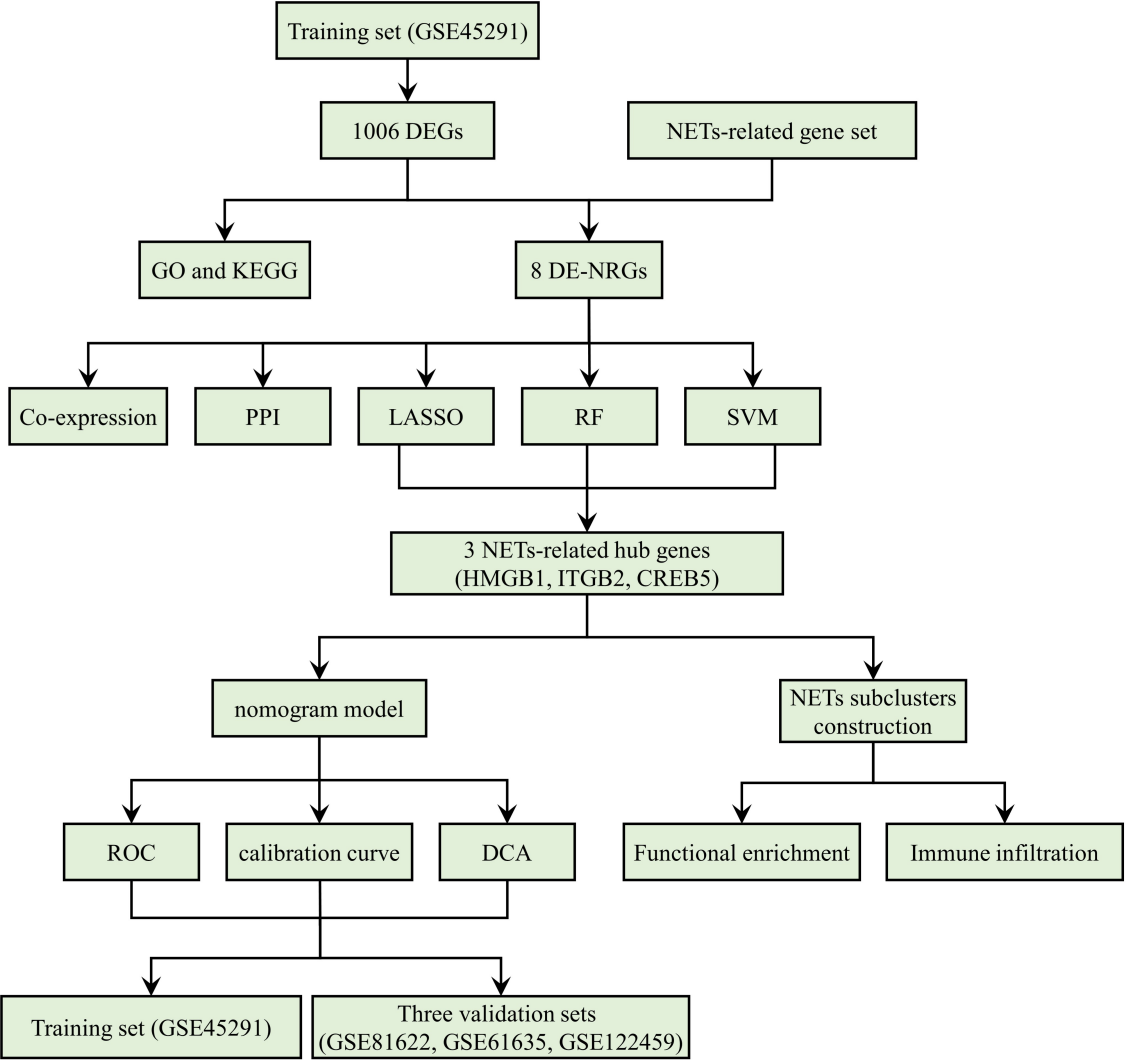
The study flow chart.

**Figure 2:**
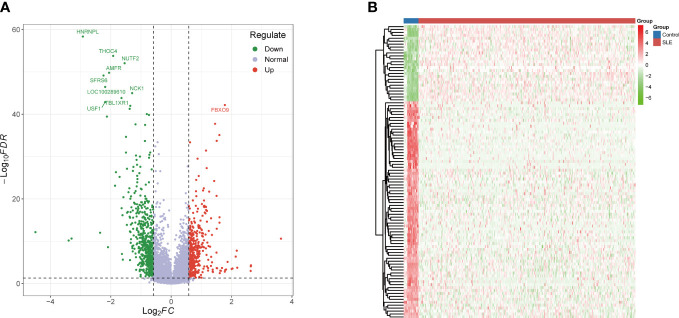
Identification of DEGs. **(A)** Volcano plot: The volcano plot was constructed using the fold change values >1.5 and adjusted P-values < 0.05. **(B)** The heatmap of the DEGs and different colors represent the trend of gene expression in different PBMCs. The top 100 genes ranked according to adjusted p-values as shown in this figure. DEGs differentially expressed genes.

**Figure 1** is a detailed flowchart of the research procedure. Based on significance criteria, a total of 1006 (393 upregulated and 613 downregulated) SLE-associated DEGs (**Supplementary Table S2**). **Figures 2A, B** depict the volcano plot and heatmap of DEGs.

### Functional annotation and pathway enrichment of DEGs

**Figure 3:**
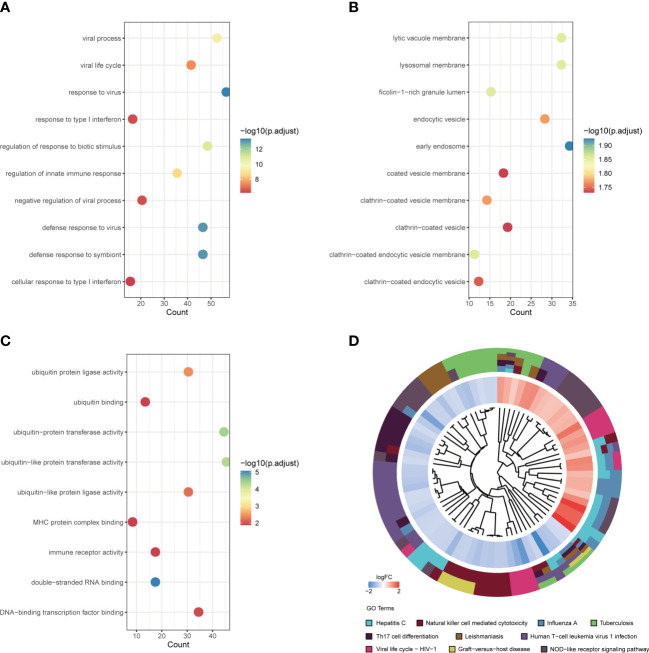
Functional annotation and pathway enrichment of DEGs. **(A)** Top 10 GO BP pathway; **(B)** Top 10 GO CC pathway; **(C)** Top 10 GO MF pathway; **(D)** Top 10 KEGG pathway. DEGs, differentially expressed genes; GO, gene ontology; BP, biological process; CC, cellular component; MF, molecular function; KEGG, Kyoto encyclopedia of genes and genomes.

According to GO-BP analysis, the DEGs were considerably increased in viral processes, viral life cycle, the response against virus and type I interferon, regulation of biotic stimulus and innate immune responses, negative viral process regulation, defense response to virus and symbiont, and cellular response to type I interferon (**Figure 3A**). The findings of the GO-CC analysis were primarily lytic vacuole membrane, lysosomal membrane, ficolin-1-rich granule lumen, early endosome, endocytic vesicle, coated vesicle membrane, and clathrin-coated vesicle, vesicle membrane, and endocytic vesicle (**Figure 3B**). The activity of ubiquitin-protein ligase, ubiquitin-binding, ubiquitin-protein transferase, ubiquitin-like protein transferase, ubiquitin-like protein ligase, MHC protein complex binding, immune receptor, double-stranded RNA binding, and DNA-binding transcription factor binding were the key outcomes of enrichment analysis in GO-MF (**Figure 3C**). The KEGG pathway analysis primarily indicated that these DEGs were associated with virus-related diseases including Hepatitis C, Natural killer cell mediated cytotoxicity, Influenza A, Tuberculosis, Th17 cell differentiation, Leishmaniasis, Human T-cell leukemia virus 1 infection, Viral life cycle – HIV – 1, Graft-versus-host disease, and NOD-like receptor signaling pathway (**Figure 3D**).

### Gene expression patterns and PPI network of DE-NRGs

**Figure 4:**
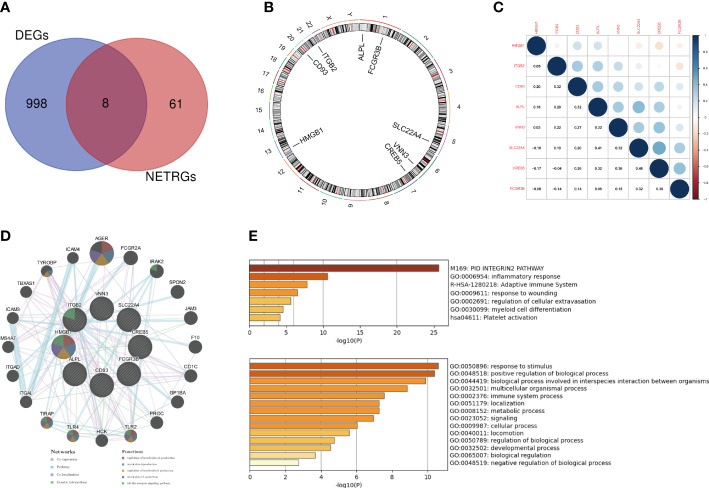
Gene expression patterns and PPI network of DE-NRGs. **(A)** The overlap of genes between DEGs and NRGs. **(B)** The location of the DE-NRGs on chromosomes. **(C)** Correlation matrix of DE-NRGs. **(D)** PPI network of DE-NRGs and its interacting proteins. **(E)** GO enrichment analysis of interacting proteins of DE-NRGs. PPI, protein-protein interaction; DEGs, differentially expressed genes; NRGs, NETs-related genes; GO, gene ontology.

The interaction of DEGs with NRGs resulted in 8 DE-NRGs (**Figure 4A**), and the location of these DE-NRGs on chromosomes was: ALPL (chr1), CD93 (chr20), CREB5 (chr7), FCGR3B (chr1), HMGB1 (chr13), ITGB2 (chr21), SLC22A4 (chr5), and VNN3 (chr6) (**Figure 4B**). The gene relationship network diagram indicated the correlation among these DE-NRGs (**Figure 4C**). Additionally, a DE-NRGs network was constructed, comprising 20 other genes including AGER, FCGR2A, IRAK2, SPON2, JAM3, F10, CD1C, GP1BA, PROC, TLR2, HCK, TLR4, TIRAP, ITGAL, ITGAD, MS4A7, ICAM3, TBXAS1, TYROBP, and ICAM4 (**Figure 4D**), which were mainly involved in PID_INTEGRIN2_PATHWAY (**Figure 4E**).

### Identification of NETs-related hub genes for SLE

**Figure 5:**
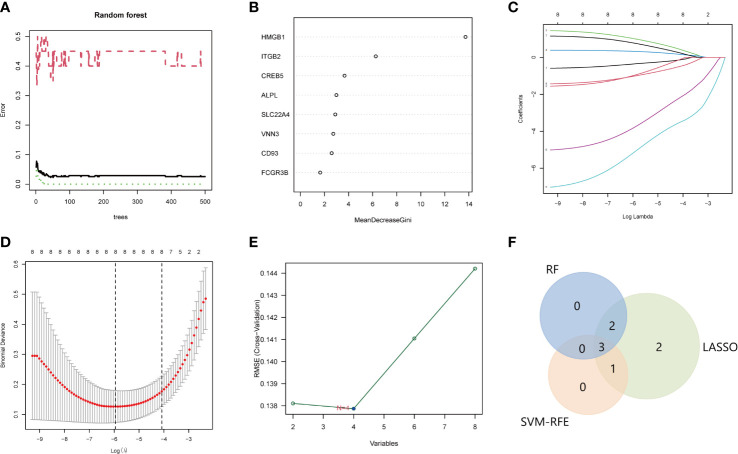
Identification of the hub genes for SLE. A-B. RF to screen candidate hub genes. **(A)** The influence of the number of decision trees on the error rate. The x-axis is the number of decision trees and the y-axis is the error rate. Green represents the SLE samples, red represents the non-SLE samples, and black represents the overall samples. **(B)** Gini importance measure. The horizontal axis represents the mean decrease Gini, and the vertical axis represents characteristic NRGs. C-D. LASSO logistic regression to screen candidate hub genes. **(C)** LASSO coefficient spectrum of eight genes enrolled and generate a coefficient distribution map for a logarithmic sequence. **(D)** The 10-fold cross-validation process was repeated 10 times to select the optimal penalization coefficient lambda. The value of lambda yielded the minimum average binomial deviance that was used to select features. **(E)** The RMSE of candidate hub genes combination of the SVM algorithm. The minimum classification error (minimal RMSE = 0.1377) in the condition of four candidate genes. **(F)** The Venn diagram shows the overlap of candidate genes among the above three algorithms. SLE, systemic lupus erythematosus; RF, random forest; NRGs, NETs-related genes; LASSO, least absolute shrinkage, and selection operator; RMSE, root-mean-square error; SVM, support vector machine.

The LASSO, SVM, and RF used DE-NRGs expression levels to discriminate SLE from healthy controls. In the RF classifier, the optimal number of trees selected was 44 as it has the lowest error rate and stability (**Figure 5A**), to obtain the dimensional importance of the DE-NRGs. Furthermore, the top 5 genes (HMGB1, ITGB2, CREB5, ALPL, and SLC22A4) based on their importance in MeanDecreaseGini result (**Figure 3B**), were selected as candidate hub genes. In LASSO logistic regression, all the DE-NRGs were recognized as candidate hub genes according to the optimal lambda = 0.002619094 (**Figures 5C, D**). The SVM model has the minimum classification error [minimal root-mean-square error (RMSE) = 0.1377] in the case of four candidate genes; HMGB1, ITGB2, CREB5, and VNN3 (**Figure 5E**). Finally, HMGB1, ITGB2, and CREB5, overlapping genes by the three algorithms, were selected as hub genes (**Figure 5F**).

### Performance of NETs-related hub genes

**Figure 6:**
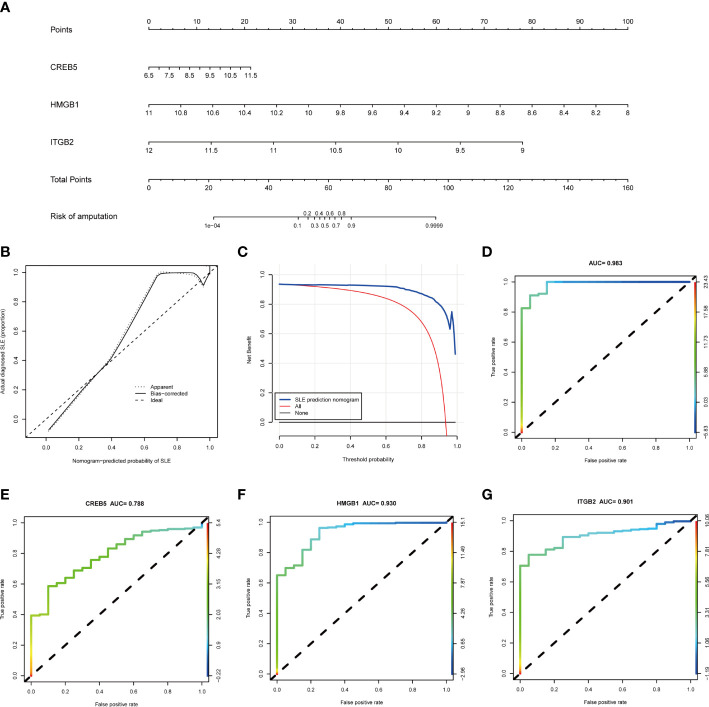
The performance of hub genes in the training set. **(A)** Construction of a nomogram for predicting the occurrence of SLE based on hub genes. **(B)** Calibration curve. The y-axis is the actual rate of SLE diagnosis and the x-axis is the predicted risk of SLE. The diagonal dotted line represents a perfect prediction by an ideal model. The solid line represents the bias−corrected performance of the nomogram, where a closer fit to the diagonal dotted line represents a better prediction. **(C)** Decision curve analysis. The blue represents the net benefit of the nomogram in the prediction of SLE occurrence. The red line represents the assumption that all people have SLE. The black line represents the assumption that all people do not have SLE. **(D)** ROC curve of the three hub genes combined diagnosis of SLE. **(E)** ROC curve of CREB5 gene diagnosis of SLE; **(F)** ROC curve of HMGB1 gene diagnosis of SLE; **(G)** ROC curve of ITGB2 gene diagnosis of SLE. SLE, systemic lupus erythematosus; ROC, receiver operating characteristic.

**Figure 7:**
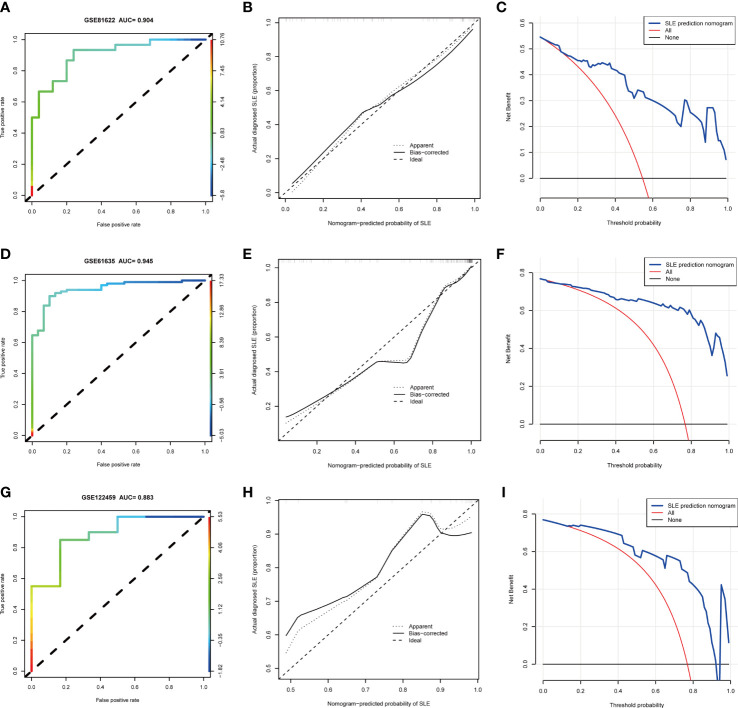
The performance of hub genes in the validation sets. **(A–C)**. GSE81622. **(D–F)**. GSE61635. **(G–I)**. GSE122459.

Based on the three NETs-related hub Genes, a nomogram model was constructed to calculate the odds of developing SLE and to further evaluate their predictive effectiveness (**Figure 6A**). The calibration curve (**Figure 6B**) and DCA (**Figure 6C**) confirmed the nomogram’s performance. A combined ROC analysis of the three hub genes was carried out with an AUC as high as 0.983 (**Figure 6D**), indicating outstanding diagnostic efficiency. Furthermore, AUCs of 0.788, 0.930, and 0.901 for CREB5, HMGB1, and ITGB2 respectively, showed their potency as useful diagnostic biomarkers (**Figures 6E–G**). In addition, the three validation datasets, GSE81622 (**Figures 7A–C**), GSE61635 (**Figures 7D–F**), and GSE122459 (**Figures 7G–I**), were utilized to independently assess this model’s performance by ROC curves, calibration curve, and DCA, and found that it performed well in the validation sets with AUCs of 0.904, 0.945, and 0.883, respectively.

### Identification of NETs-related sub-clusters in SLE

**Figure 8:**
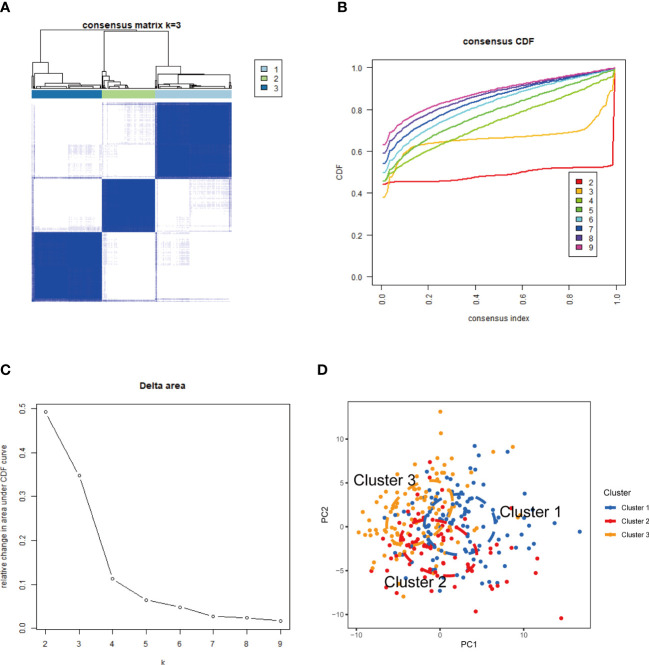
NETs-related molecular clusters identification in SLE. **(A)** Consensus clustering matrix when k = 3; **(B)** Consensus clustering CDF for k = 2–9. **(C)** Relative change in area under CDF curve for k = 2–9. **(D)** PCA was used to verify the three distinct subgroups divided by consensus clustering. SLE, systemic lupus erythematosus; CDF, cumulative distribution function; PCA, principal component analysis.

The CREB5, HMGB1, and ITGB2 expression profiles were used to group the 292 SLE samples *via* a consensus clustering algorithm. The k value of three (k = 3) gave the most stable cluster numbers, based on the above criteria (**Figures 8A–C**). As the PCA plot indicates, gene expression patterns were specific across the sub-clusters (**Figure 8D**).

### Functional distinctions among the sub-clusters

**Figure 9:**
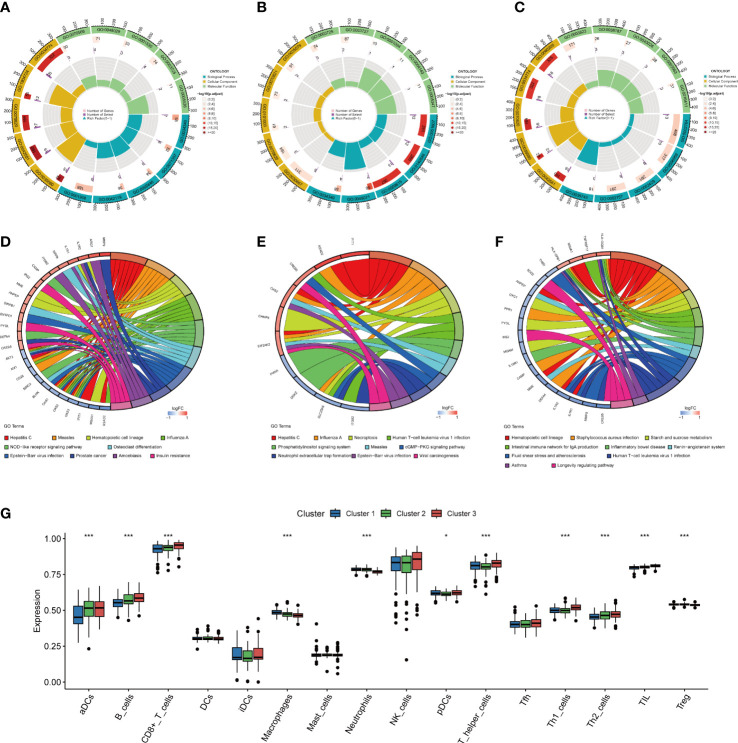
Functional enrichment and immune cell infiltration analyses among three NETs subgroups. **(A–C)**. GO enrichment analysis of DEGs highly expressed in cluster 1 **(A)**, cluster 2 **(B)**, and cluster 3 **(C)**. **(D–F)**. KEGG enrichment analysis of DEGs highly expressed in cluster 1 **(D)**, in cluster 2 **(E)**, and in cluster 3 **(F)**. **(G)** Box plot showing the relative abundances of immune cells among three clusters, *P < 0.05, ***P < 0.001. NETs, neutrophil extracellular traps; GO, gene ontology; KEGG, Kyoto encyclopedia of genes and genomes.

A total of 107, 25, and 38 DEGs, highly expressed in cluster1 (**Supplementary Table S3**), cluster2 (**Supplementary Table S4**), and cluster3 (**Supplementary Table S5**) respectively were identified. As GO analysis indicates, the cluster 1 highly expressed DEGs were enriched in cell killing (GO:0001906), neutrophil activation (GO:0042119), neutrophil-mediated immunity (GO:0002446), cellular response to type I interferon (GO:0071357), and response to type I interferon (GO:0034340) (**Figure 9A**), and the cluster 2 highly expressed DEGs were enriched in response to type I interferon (GO:0034340), response to the virus (GO:0009615), defense response to symbiont (GO:0140546), negative regulation of viral genome replication (GO:0045071), and defense response to the virus (GO:0051607) (**Figure 9B**), and the DEGs highly expressed in cluster 3 were enriched in CD4-positive, alpha-beta T cell cytokine production (GO:0035743), immune response-activating signal transduction (GO:0002757), immune response-activating cell surface receptor signaling pathway (GO:0002429), humoral immune response (GO:0006959), and immune response-regulating signaling pathway (GO:0002764) (**Figure 9C**).

The KEGG enrichment analysis revealed that cluster 1 highly expressed DEGs were mostly prevalent in Hepatitis C, Measles, Hematopoietic cell lineage, Influenza A, NOD-like receptor signaling pathway, Epstein-Barr virus infection, Prostate cancer, Amoebiasis, Osteoclast differentiation, and Insulin resistance (**Figure 9D**), and that of cluster 2 were mostly enriched in Hepatitis C, Influenza A, Necroptosis, Human T-cell leukemia virus 1 infection, Phosphatidylinositol signaling system, Epstein-Barr virus infection, Measles, cGMP-PKG signaling pathway, Neutrophil extracellular trap formation, and Viral carcinogenesis (**Figure 9E**). Cluster 3 highly expressed DEGs were mostly associated with Staphylococcus aureus infection, Hematopoietic cell lineage, production of intestinal immune network for IgA, Starch and sucrose metabolism, Human T-cell leukemia virus 1 infection, Inflammatory bowel disease, Renin-angiotensin system, Fluid shear stress and atherosclerosis, Asthma, and Longevity regulating pathway (**Figure 9F**).

### Differentiation of immune infiltration characteristics among the sub-clusters

The immune infiltration analysis indicated the presence of an altered immune microenvironment between the sub-clusters (**Figure 9G**). Cluster1 exhibited the highest proportions of macrophages and neutrophils, whereas pDCs, aDCs, and CD8+ T, B, Th1, T helper, Th2, and TIL cells were abundant in Cluster3.

## Discussion

SLE is an autoimmune disorder comprising a complex network of immune-inflammatory pathological mechanisms. The NETs functions have been associated with the development and progression of SLE, however, its potential mechanisms that contribute to SLE at the genetic level have not been investigated. This investigation explores the molecular characteristics of NRGs in SLE based on bioinformatics analyses and machine learning, and identify three reliable biomarkers and three molecular clusters of SLE.

Here GEO database was utilized to elucidate the gene expression levels of healthy controls and SLE patients, and 1006 DEGs, including 393 upregulated and 613 downregulated genes were identified. The DEGs were then annotated using the research results on function-related enrichment. As results indicate, these genes were most prevalent in different viral infections and their associated with the immune response pathways. Previous research has revealed that pathogens, especially viruses, are important environmental triggers of SLE (44), which reinforce our results. Previous studies have highlighted the significance of NETs in the pathophysiology of SLE (8–10). In order to investigate the molecular properties of NRGs in SLE, the intersection of DEGs and NRGs was carried out, and 8 DE-NRGs were subsequently obtained. Further analysis of these 8 DE-NRGs revealed that most of them had significant co−expression tendencies, which indicated that these genes may work together in the pathogenesis of SLE. Notably, the PPI analysis revealed that the DE-NRGs interacting genes were mainly involved in the PID_INTEGRIN2_PATHWAY. The Beta2-integrins, comprising a common Beta2 (CD18) subunit complexed with rare subunits (CD11a-d), including LFA-1 (leukocyte function antigen 1, CD11a/CD18), Mac-1 (CD11b/CD18, aMb2 or complement receptor 3), alphaXbeta2 (CD11c/CD18) and alphaDbeta2 (CD11d/CD18), have significant activity in immigration by endothelial and chemotaxis, leukocyte adhesion, and immune and inflammatory reactions (45). Among them, LFA-1 and Mac-1 are the most studied and have been reported to be linked with SLE pathogenesis (46, 47). For instance, loss of LFA-1 activity protects against the development of lupus in mouse models whereas its overexpression causes lupus-like syndrome (48). Furthermore, in a spontaneous SLE model, the MRL/lpr mice, Mac-1 reduction causes severe glomerulonephritis that is related to an increase in neutrophil infiltration in the kidneys (49). However, how these genes contribute to SLE and its clear mechanisms need to be discussed with functional studies in the future.

With the rapid advancements of artificial intelligence (AI), machine learning algorithm, as an important branch, can better discriminate and have higher-dimensional feature data, and has been widely used for hub gene identification and screening (50). In this investigation, the predictive performance of three selected machine learning classifiers (RF, SVM, and LASSO) was integrated based on DE-NRGs expression profiles. Finally, CREB5, HMGB1, and ITGB2 were selected as hub genes, whose diagnostic values for SLE have been validated in the training and three validation sets.

CREB5 (CAMP Responsive Element Binding Protein 5), belongs to the CRE (cAMP response element)-binding protein family and encodes a transcription activator in eukaryotic cells (51). CREB5-associated pathways include PI3K-Akt and Toll-like receptor signaling pathways (52). The PI3K-AKT pathway regulates various physiological responses and is critical for lymphocyte development and optimal immune responses (53, 54). In the lupus MRL/lpr mouse model, it was found that excessive PI3K/Akt pathway activation leads to the severity of inflammation and fibrosis in the kidney while its inhibition reduces glomerulonephritis and prolonged life span (55–57). The toll-like receptors (TLRs) respond to molecular patterns associated with microbes such as bacteria and viruses, stimulating innate and acquired immunity as well as an inflammatory response and cytokines upregulation (58). Several studies suggest that the signaling pathway has essential activity in the pathogenesis of autoimmune diseases including SLE (59, 60). Therefore, we speculate that CREB5 may participate in the pathogenesis of SLE with the involvement of PI3K-Akt and Toll-like receptor signaling pathways and serve as a potential therapeutic target. However, the relevance of CREB5 in SLE has not been documented and further research is required. HMGB1 (High Mobility Group Box 1), encodes a protein that belongs to the High Mobility Group-box superfamily and exerts pro-inflammatory effects in SLE through both innate and adaptive immunity (61, 62). SLE individuals have aberrant apoptotic debris removal, and apoptotic or necrotic cells release HMGB1 as a damage-associated molecular pattern (DAMP), causing tissue and organ damage and dysfunction (61). HMGB1 has been associated with multiple disease phenotypes in SLE, including lupus nephritis, neuropsychiatric lupus, and skin lesions (61, 63). ITGB2 (Integrin Subunit Beta 2) gene encodes an integrin beta chain (CD18) and combines with multiple different alpha chains to form different Beta2-integrins (45), as mentioned earlier. Thus, the NETs hub genes have potential roles in promoting the occurrence and development of SLE, and some specific pathways can act as therapeutic targets for controlling SLE.

In addition, unsupervised cluster analysis illustrated different NETs regulation patterns in SLE patients based on hub gene expression, and three distinct clusters were identified. Interestingly, functional enrichment was performed among three NETs subgroups, and the data showed that the DEGs highly expressed in cluster 1 were enriched in innate immune response pathways, such as neutrophil activation, neutrophil-mediated immunity, response to type I interferon, and so on, whereas that of cluster 3 were enriched in adaptive immune response pathways. Moreover, immune infiltration analysis also revealed that innate immune cells including macrophages and neutrophils were significantly upregulated in cluster 1, in contrast, cluster 3, which was dominated by upregulated T and B cells of the adaptive immunity. It is well known that SLE is characterized by its clinical and therapy heterogeneity, with a wide range of clinical manifestations and drug responses reflecting its complex etiopathogenesis, which means that stratified management planned accordingly may provide better outcomes. Consequently, the data of this investigation may provide potential biological insights into the different clinical phenotypes and a rationale for the stratified therapy of patients.

This investigation has several limitations that should be taken into consideration. Firstly, the study only relies on bioinformatics analyses, and further experimental validation is needed to confirm the results. Additionally, the findings were obtained from a relatively small sample size of SLE patients, and larger cohorts are necessary to provide more robust and reliable results. Moreover, the SLE diagnostic model developed in this study requires further evaluation and external validation before its clinical application can be considered. Finally, this investigation only focused on gene expression data, and future studies should also explore epigenetic, proteomic, and metabolomic alterations in SLE pathogenesis.

In conclusion, this research is the first as per our knowledge to explore the molecular characteristics of NRGs in SLE, identify three potential biomarkers, HMGB1, ITGB2, and CREB5, and three distinct clusters based on these hub biomarkers. These findings may assist in diagnosing and managing SLE with the ultimate objective of improving patient outcomes.

## Data availability statement

The original contributions presented in the study are included in the article/**Supplementary Material**. Further inquiries can be directed to the corresponding author.

## Author contributions

XD and HL designed the study. HL, XZ, JS, XF, LY, JF, JR, and RZ performed data analysis. HL drafted the manuscript. XD revised the manuscript. All authors read and approved the final manuscript. All authors contributed to the article and approved the submitted version.
